# Connection Length Controlled Sound Speed and Thermal Conductivity of Hybrid Metalcone Films

**DOI:** 10.1021/acs.nanolett.4c03741

**Published:** 2025-02-10

**Authors:** Md Shafkat Bin Hoque, Rachel A. Nye, Saman Zare, Stephanie Atkinson, Siyao Wang, Andrew H. Jones, John T. Gaskins, Gregory N. Parsons, Patrick E. Hopkins

**Affiliations:** † Department of Mechanical and Aerospace Engineering, University of Virginia, Charlottesville, Virginia 22904, United States; ‡ Department of Chemical and Biomolecular Engineering, North Carolina State University, Raleigh, North Carolina 27606, United States; § Laser Thermal, Charlottesville, Virginia 22902, United States; ∥ Department of Materials Science and Engineering, University of Virginia, Charlottesville, Virginia 22904, United States; ⊥ Department of Physics, University of Virginia, Charlottesville, Virginia 22904, United States

**Keywords:** Molecular layer deposition, thermal conductivity, vibrational lifetime, metalcone

## Abstract

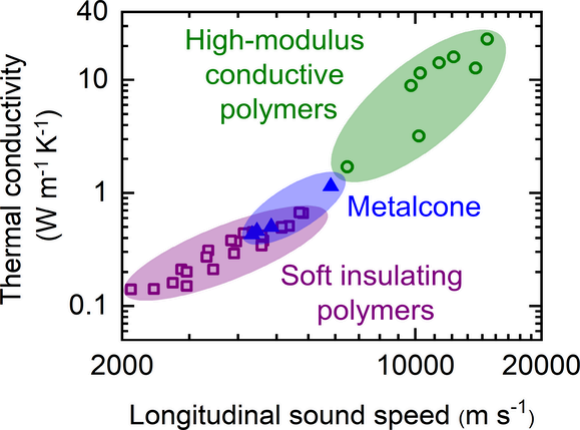

The multifaceted applications of polymers are often limited by their thermal conductivity. Therefore, understanding the mechanisms of thermal transport in polymers is of vital interest. Here, we leverage molecular layer deposition to grow three types of hybrid metalcone (i.e., alucone, zincone, and tincone) films and study their thermal and acoustic properties. The thermal conductivity of the hybrid polymer films ranged from 0.43 to 1.14 W m^–1^ K^–1^. Using kinetic theory, we trace the origin of thermal conductivity difference to sound speed change, which is dictated by the connection length within the films. Changing the connection length has negligible impacts on volumetric heat capacity and vibrational lifetimes. Our findings provide means to improve the thermal conductivity of organic, hybrid, and inorganic polymer films.

The thermal, acoustic, and optical properties of polymers have been widely studied in literature due to their applications in electronic devices as flexible substrates, encapsulation layers, and insulating materials.^1−6^ The thermal conductivity of polymers can span across 3 orders of magnitude, ranging from 0.2 W m^–1^ K^–1^ in PMMA^7^ to 23 W m^–1^ K^–1^ in Zylon HM.^8^ Length and type of interactions between the polymer chains generally control the thermal conductivity of polymers.^9−13^ However, establishing a clear physical picture of thermal transport in polymers has been a challenge. Study of new types of polymers with different connection lengths can shed light on this aspect.

Only recently, molecular layer deposition (MLD)^5^ has been successful in growing different types of stable hybrid organic–inorganic metalcone polymers. MLD uses a bifunctional organic monomer and multifunctional inorganic precursor to grow the hybrid metalcone films.^14^ In addition to the usual applications of polymers, hybrid polymers are also useful in organic light emitting diodes and lithium/sodium ion batteries.^15−17^ Use of metalcone films in these devices has resulted in improved lifetimes and stability. However, due to the relatively new age of the metalcone films, their thermal and acoustic properties remain unexplored.

In this study, we characterize the thermal and acoustic properties of three different types of amorphous metalcone films (alucone, zincone, and tincone-grown via MLD). The three metalcone films each possess a different connection length (i.e., the distance between the metal atoms in the chain) based on growth. This is directly determined by the length of the molecules used during MLD. We use steady-state thermoreflectance (SSTR)^18−20^ and time-domain thermoreflectance (TDTR)^21−23^ to measure the thermal conductivity and longitudinal sound speed of the films, respectively. Our measurements reveal that the connection lengths control the longitudinal sound speed of the films. The sound speed, in turn, dictates the thermal conductivity of the metalcone films. We further verify the findings by measuring thermal conductivity, longitudinal sound speed, volumetric heat capacity, and lifetime of the vibration modes of a short connected and long connected alucone film. Our study opens up new pathways for tuning the thermal and acoustic properties of hybrid polymer films by manipulating connection lengths.

The metalcone films are deposited via MLD using various metal–organic precursors and ethylene glycol (EG) as the coreactants. The connection length is directly determined by the length of the molecule we use during MLD. Alucone is deposited from trimethylaluminum (TMA) and EG. Zincone is deposited from diethyl zinc (DEZ) and EG.^24^ Tincone is deposited from tetrakis (dimethylamido) tin (TDMASn) and EG. Each precursor is handled only in nitrogen-ambient before being installed on the home-built cylindrical or spherical MLD reactors.^5,25^ Precursors are heated as follows to achieve sufficient vapor pressure for deposition: TDMASn was set to 65 °C. TMA was set to room temperature. DEZ was set to room temperature, and EG was set to 80 °C for alucone and 70 °C for tincone and zincone. The deposition is carried out at 100 °C and 400 mTorr. The silicon (Si) substrates are cleaned in a piranha solution of 1:1 volume ratio H_2_O_2_/H_2_SO_4_ for 15 min before use. The structures of the metalcone films are shown in Figure 1. The flexibility of the organic components gives rise to an amorphous phase, as characterized by X-ray diffraction (Supporting Figure S1). The thicknesses of the amorphous metalcone films range from 1 to 156 nm.

**Figure 1:**
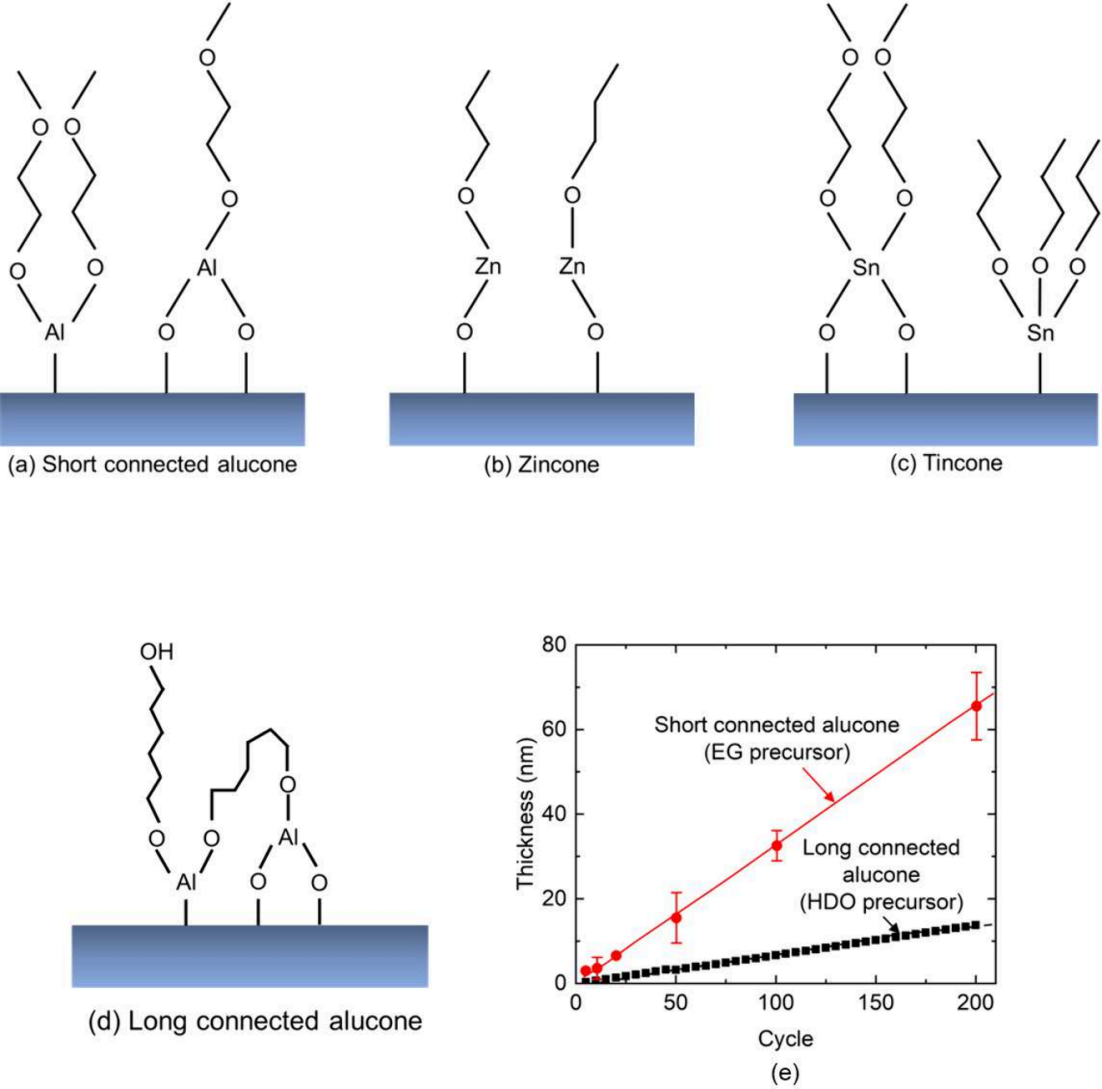
Structures of the (a) short connected alucone, (b) zincone, (c) tincone, and (d) long connected alucone films. The different connection lengths of the alucone films are visually illustrated in Supporting Figures S2 and S3. (e) Thickness vs cycle for the short and long connected alucone films measured by ex situ and in situ ellipsometry, respectively.

To study the effects of connection length on the thermal and acoustic properties of the metalcone films, we also deposit alucone films from TMA and 1,6-hexamethylenediol (HDO). While alucone films from TMA/EG are common, HDO represents a more unique and much less studied reactant. The alucone film grown from HDO precursors possesses longer connection lengths compared to that of EG as discussed in the next section. As a result, we define the HDO alucone films as long connected in this study.

We characterize the connection lengths in the metalcone films by the phenomenon of double reactions (DRs).^25,26^ When DRs occur, both reactive groups of the homobifunctional precursors react with the growth surface during the same half-cycle without providing a new site for continued growth. DRs are more common for more flexible molecules (i.e., with longer aliphatic hydrocarbon chains). Among the metalcone films, tincone is expected to undergo a larger number of DRs during deposition compared to alucone because the tin precursor can bond with more organic ligands (4 and 3 for tincone and alucone, respectively). Increased DRs will lead to increased interactions between adjacent molecules and chains in a film, thereby increasing connection lengths in tincone compared to the alucone films.

Moreover, for the alucone films, the DRs are expected to be different based on the precursors (i.e., EG and HDO) used. EG is relatively short and is expected to have few DRs and give rise to relatively short connection lengths of the alucone films. On the other hand, the longer chain HDO precursor is expected to undergo more frequent DRs, thus resulting in more cross-linked or tangled chains during deposition and a long connected film. The structure of a long connected alucone film is shown in Figure 1d.

Figure 1e plots film thickness as a function of the atomic layer deposition cycle for both alucone films (TMA/EG and TMA/HDO), as measured by spectroscopic ellipsometry. The growth rates correspond to 0.3 and 0.07 nm/cycle for alucone deposited using EG and HDO, respectively, consistent with previous results.^27^ The higher growth rate for the less flexible precursor (i.e., EG compared to HDO) is consistent with fewer expected double reactions for EG, as observed in many MLD processes.^5,26,28^ The growth rate data shown in Figure 1e support a difference in connection length within the films.

The thermal and acoustic properties of the metalcone films are characterized by the laser-optical thermoreflectance techniques SSTR and TDTR, respectively. We use 1/e^2^ pump and probe diameters of ∼20 μm for SSTR, whereas for TDTR, the diameters are ∼20 and 11 μm, respectively. Additional details of the techniques can be found in previous publications.^18−20,23,30^ To convert the optical energy of the lasers into thermal energy, we deposit a thin aluminum film atop the samples via electron beam evaporation prior to the thermoreflectance measurements.^31^

Figure 2a shows a schematic diagram of the sample geometry. As most of the metalcone films have thicknesses less than 50 nm, measuring thermal conductivity directly can be challenging. Therefore, we measure the total thermal resistance (*R*) across the entire sample geometry via SSTR. The total thermal resistance can be expressed via the following series resistor model:^31^
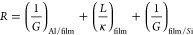
1where *G*, κ, and *L* represent thermal boundary conductance, thermal conductivity, and thickness of the metalcone films, respectively. Figure 2b shows the measured thermal resistances of the films as a function of the thickness. As exhibited here, thermal resistance linearly increases with film thickness. For such cases, thermal conductivity can be extracted from a linear fit to the thermal resistance as a function of film thickness. Here, the inverse of the slope (Δ*R*/Δ*L*)^−1^ provides the thermal conductivity of the films.^32^ Using this methodology, we determine the thermal conductivity of the metalcone films, as tabulated in Table 1.

**Figure 2:**
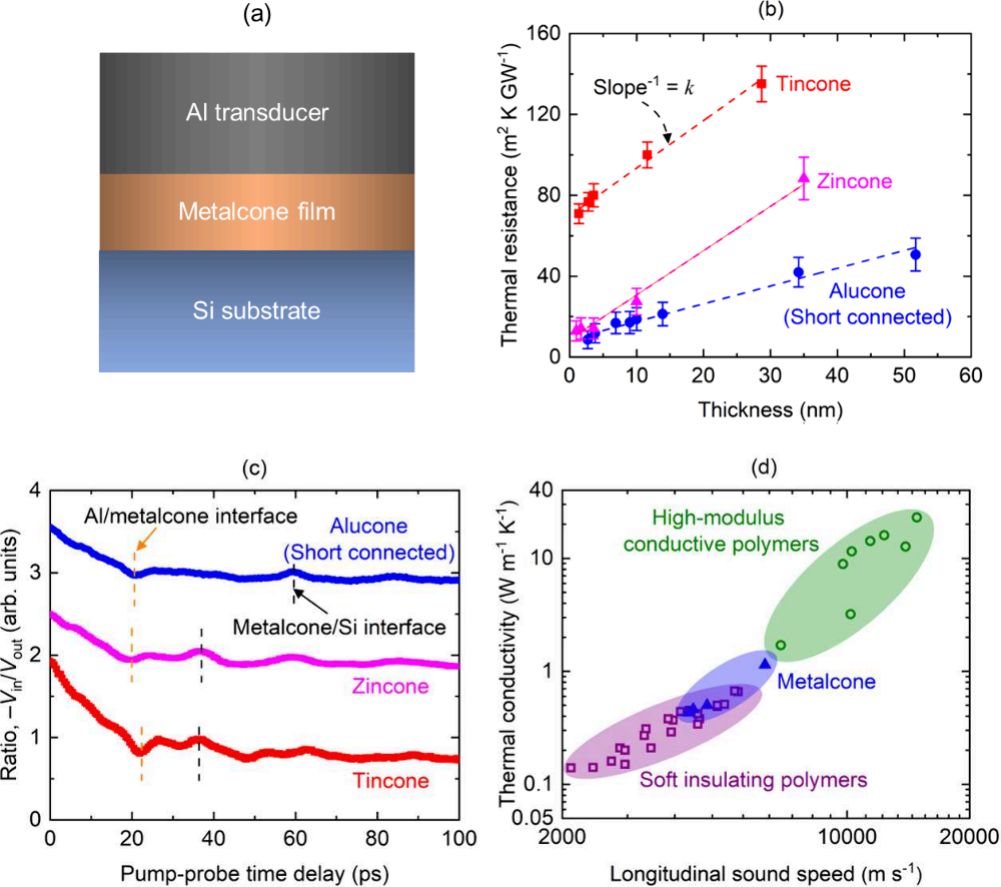
(a) Schematic diagram of the sample geometry. (b) Thermal resistance as a function of alucone (short connected), zincone, and tincone film thickness. (c) Picosecond acoustic response of TDTR measurements for a 120, 35, and 29 nm alucone (short connected), zincone, and tincone film, respectively. (d) Thermal conductivity vs longitudinal sound speed for a wide range of polymers. The data for the soft insulating polymers and high-modulus conductive polymers (hollow symbols) are taken from refs (7), (8), and (29).

**Table 1:** Thermal Conductivity and Longitudinal Sound Speed of the Metalcone Films

Metalcone films	Thermal conductivity (W m^–1^ K^–1^)	Longitudinal sound speed (m s^–1^)
Alucone (EG precursor)	1.14 ± 0.18	6288 ± 324
Alucone (HDO precursor)	0.50 ± 0.08	4532 ± 230
Zincone	0.46 ± 0.06	4192 ± 401
Tincone	0.43 ± 0.07	4080 ± 297

We measure the longitudinal sound speed of the metalcone films via picosecond acoustics^30,33−37^ using TDTR. Figure 2c shows the picosecond acoustic response of the metalcone films. As exhibited in Table 1, alucone films (EG precursor) have the highest sound speed, whereas tincone films have the lowest. We posit that the sound speed difference between the two metalcone films stems from the difference in connection lengths. As sound speed is an indicator of stiffness or elastic modulus of a material,^34^ the connection length is also changing the stiffness of the metalcone films.

According to the simple kinetic theory, κ = 1/3*Cv*^2^τ, where *C*, *v*, and τ represent volumetric heat capacity, sound speed, and lifetime of the vibrational modes, respectively.^33,38^ This relation is powerful in describing the key parameters that govern thermal conductivity: heat capacity, sound speed, and vibrational lifetimes. This theory is widely used to study thermal transport in amorphous materials and forms the basis of multiple minimum thermal conductivity models.^33,38−43^ Using this equation, the thermal conductivity difference among the metalcone films can be quantitatively explained by the difference in sound speed. This provides evidence that instead of volumetric heat capacity or lifetimes, sound speed dictates the thermal conductivity of the metalcone films. A similar trend has been observed in the literature^7,8,29^ for other polymers as illustrated in Figure 2d. This figure also shows that the metalcone films can bridge an important gap between the soft, insulating polymers and high-modulus, conductive polymers.

To further show the impact of connection lengths on the sound speed and thermal conductivity of the metalcone films, we measure κ, *C*, *v*, and τ of a short (EG precursor) and a long connected (HDO precursor) alucone film. The TDTR multifrequency approach^44,45^ is used to simultaneously measure the thermal conductivity and volumetric heat capacity of 120 nm short and 156 nm long connected alucone films. This approach uses different modulation frequencies and thermal penetration depths to alter sensitivities to volumetric heat capacity and thermal conductivity. The crossover point of the frequencies provides the volumetric heat capacity and thermal conductivity, as shown in Figure 3a. The volumetric heat capacity of the alucone film remains the same for both cases: 2.0 ± 0.3 MJ m^–3^ K^–1^. The thermal conductivity, on the other hand, drops from 1.05 ± 0.15 W m^–1^ K^–1^ for the short connected film to 0.50 ± 0.08 W m^–1^ K^–1^ for the long connected film (Table 1). The longitudinal sound speed also changes from 6288 ± 324 m s^–1^ for the short connected film to 4532 ± 230 m s^–1^ for the long connected film. The picosecond acoustic response of the long connected alucone film is shown in Supporting Figure S4. The different thicknesses of the short and long connected alucone films are not expected to have an impact on the thermal conductivity and sound speed measurements.^5,35^

**Figure 3:**
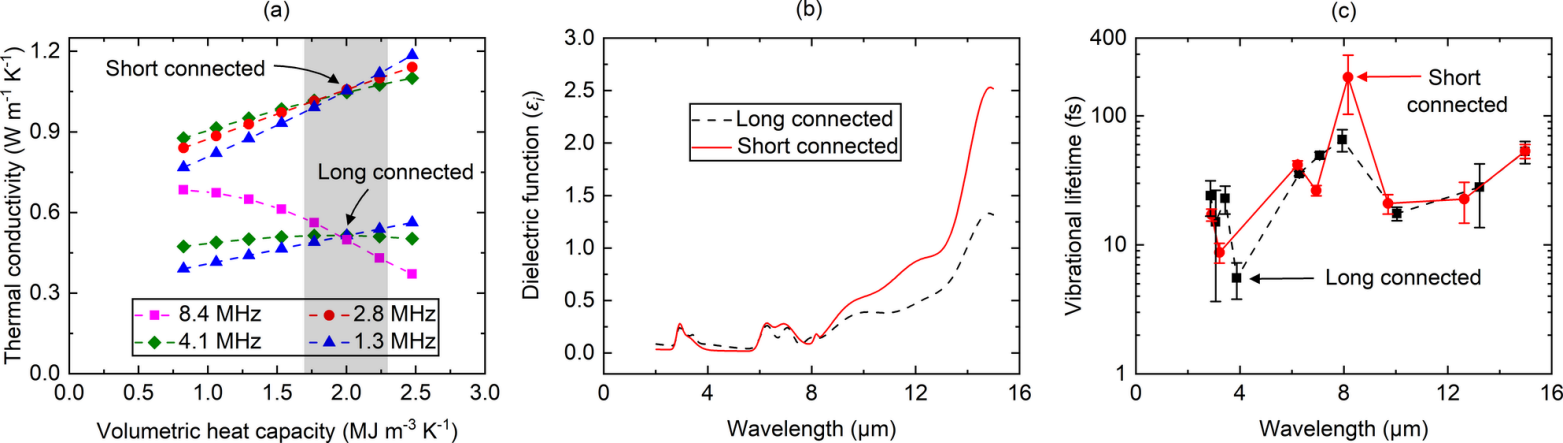
(a) Thermal conductivity and volumetric heat capacity of the short and long connected alucone film. (b) Imaginary component of the dielectric function for the two cases as a function of wavelength. (c) Lifetime of the vibrational modes in alucone films. The uncertainty associated with the lifetime measurements are ∼10%.

The lifetimes associated with each vibrational mode in the alucone films are found from fitted oscillator models on ellipsometric data collected using IR-VASE (IR-VASE Mark II, J.A. Woollam Company). The ellipsometric data are acquired in the spectral range of 666–5000 cm^–1^ (2–16 μm) with a resolution of 8 cm^–1^. The raw ellipsometric data (Ψ and Δ) are processed using multiple Gaussian oscillators to determine the dielectric function of the alucone film. The parameters of each Gaussian oscillator, including amplitude, energy centroid, and broadening, are optimized to fit the model to the collected ellipsometric data (see Supporting Information). Figure 3b shows the imaginary component of the dielectric function as a function of wavelength for short and long connected alucone films. The optical lifetimes for the identified modes are determined by taking the reciprocal of the broadening parameter associated with each Gaussian oscillator. As the IR-VASE measurements extend from 2 to 16 μm, the energies that are probed in these measurements are higher than that of typical thermal modes.^46^ However, the longer wavelengths of the IR VASE measurements will probe the higher energies of the room temperature Bose Einstein distribution, thus providing information on the scattering rates of the higher energy thermalized vibrations in the metalcones; these higher energy vibrational modes scatter in the thermal distribution of vibrational energies, and directly contribute to thermal transport.^47^ Raman and other high frequency vibrational lifetime measurements have shown that we can monitor the scattering rates of these high frequency modes to serve as an indicator of vibrational model scattering and thermal transport.^48−53^ Thus, while the entire infrared spectrum of IR-VASE measurements is not related to the thermal vibrational modes that contribute to thermal conductivity, the longer wave region of this spectrum serves as an indicator of how thermal vibrational scattering rates are changing and impacting thermal conductivity in the metalcones.

In Figure 3c, we plot the derived lifetimes of the vibrational modes for the two cases. The observed lifetimes are smaller than the inverse of the frequency (Tables S1 and S2). This indicates excessive scattering of vibrational modes. Such scattering is consistent with the modes observed in amorphous materials (e.g., diffusons).^33,54−59^ The lifetimes are nearly identical for short and long connected films at all wavelengths, with one exception at 8.2 μm. The weighted average of lifetimes for the short connected film is 41 ± 4 fs, whereas for the long connected film, it is 38 ± 5 fs. This conclusively shows that the thermal conductivity difference between the two films originates from the sound speed difference, not volumetric heat capacity or lifetimes. Our results thus provide a clear picture of thermal transport in hybrid metalcone films. The thermal and acoustic properties reported here can be useful when incorporating metalcone films in electronic, photonic, and optoelectronic devices.

In summary, we investigate the thermal and acoustic properties of alucone, zincone, and tincone films grown via MLD. Alucone possesses the highest thermal conductivity, while tincone possesses the lowest. Connection length dictates the thermal transport of the hybrid metalcone films. By manipulation of the connection length and hence sound speed, the thermal conductivity of the polymer films can be changed. Specifically, we find that films with longer connection lengths have lower thermal conductivity. We further verify our conclusion by measuring the volumetric heat capacity and vibrational lifetime of a short and long connected alucone film. The thermal transport mechanisms depicted in this study can also be applied to organic and inorganic polymer films.

## Supporting Information Available

Details of metalcone deposition conditions, film thickness and X-ray diffraction (XRD) characterizations, visual illustration of different connection lengths of the alucone film, picosecond acoustics of long connected alucone films, and infrared spectroscopic ellipsometry of alucone films (PDF)
