# Supplementary material for: Connection Length Controlled Sound Speed and Thermal Conductivity of Hybrid Metalcone Films
Source: Nano Lett. 2025 Feb 10;25(7):2594–9. doi: 10.1021/acs.nanolett.4c03741 (PMC11849029; doi:10.1021/acs.nanolett.4c03741)
Supplement: Supplementary file 1 — nl4c03741_si_001.pdf [file nl4c03741_si_001.pdf]

Supporting Information: Connection length controlled sound speed and thermal conductivity of hybrid metalcone films

Md Shafkat Bin Hoque[a], Rachel A. Nye[b], Saman Zare[a], Stephanie Atkinson[b], Siyao Wang[b], Andrew H. Jones[c], John T. Gaskins[c], Gregory N. Parsons[b], and Patrick E. Hopkins[a,d,e]*

[a]Department of Mechanical and Aerospace Engineering, University of Virginia, Charlottesville, Virginia 22904, USA

[b]Department of Chemical and Biomolecular Engineering, North Carolina State University, Raleigh, North Carolina 27606, United States

[c]Laser thermal, Charlottesville, Virginia 22902, USA

[d]Department of Materials Science and Engineering, University of Virginia, Charlottesville, Virginia 22904, USA

[e]Department of Physics, University of Virginia, Charlottesville, Virginia 22904, USA

*Email: phopkins@virginia.edu

S1. Metalcone deposition conditions

During metalcone deposition, purified nitrogen (N_2 , 99.999%, Arc3 Gases) is used as the carrier and purge gas. In the cylindrical reactor, tincone is deposited from TDMASn/EG following (10 s chamber evacuation/3 s TMDASn dose/60 s N_2 purge)/(0.3 s EG dose/60 s N_2 purge) and alucone is deposited from TMA/EG following (0.2 s TMA dose/60 s N_2 purge)/(0.2 s EG dose/60 s N_2 purge). In the spherical chamber, alucone is deposited from TMA/HDO following (0.4 s TMA dose/45 s N_2 purge)/(30 s chamber evacuation/8 s HDO dose/45 s N_2 purge).

S2. Film thickness characterizations

Metalcone film thickness is measured with ellipsometry either in situ (Film Sense FS-1 multiwavelength ellipsometer) or ex situ (J. A. Woollam Co. alpha-SE spectroscopic ellipsometer) at an incidence angle of $\sim$70° relative to the surface normal. In situ data is collected at 436, 521, 599, and 638 nm while ex situ data is collected from 300 to 900 nm. Thickness is determined from a Cauchy model available with each ellipsometer's software package. Uncertainty in film thickness is generally a few percent.

S3. X-ray diffraction (XRD) characterizations

To characterize the structure of the as-deposited metalcone films, we perform XRD on tincone and alucone (HDO precursor). Figure S1 reveals the amorphous phase of the films. The only visible peaks on each metalcone spectrums are attributed to the underlying Si substrate (i.e., Si(400) at 69° and Si(331) at 75°).[1] The amorphous phase is expected for the metalcone films due to the low deposition temperatures.[2,3]

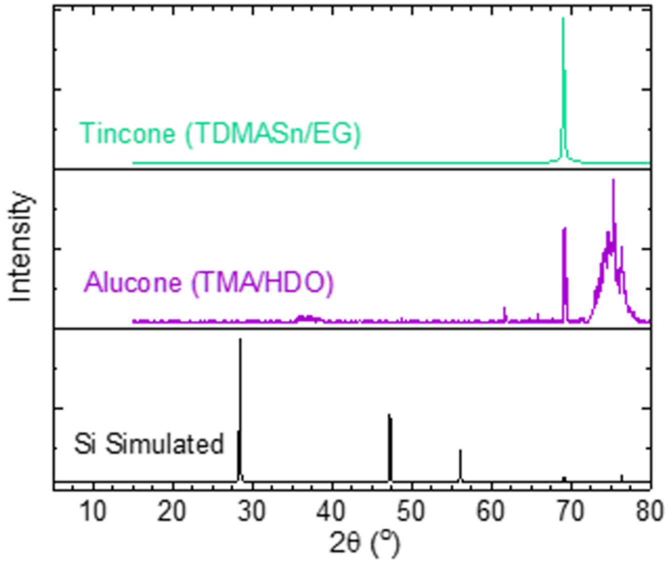

Figure S1: XRD characterizations for tincone and alucone (HDO precursor) metalcone films.

S4. Visual illustration of different connection lengths of the alucone film

The MLD process consists of a sequence of two reactions performed within a cycle, where each reaction reaches saturation and is therefore self-limiting. Because each reaction prepares the surface for the following reaction, the overall process itself can continue indefinitely.

The inhibition is directly evident by the overall smaller growth per cycle for the HDO vs EG. For both the HDO and EG reactants, the growth is expected to continue at a constant growth per cycle. However, because the HDO can bend over and block multiple reactive sites, the net growth per cycle for the HDO case is expected to be less than for the EG, which is shown in Figure 1(e).

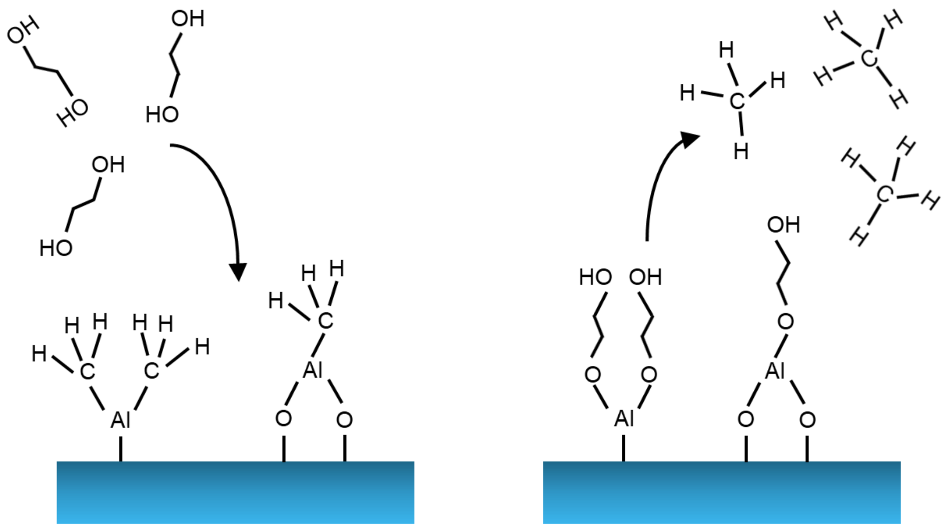

Figure S2: Short connected alucone film

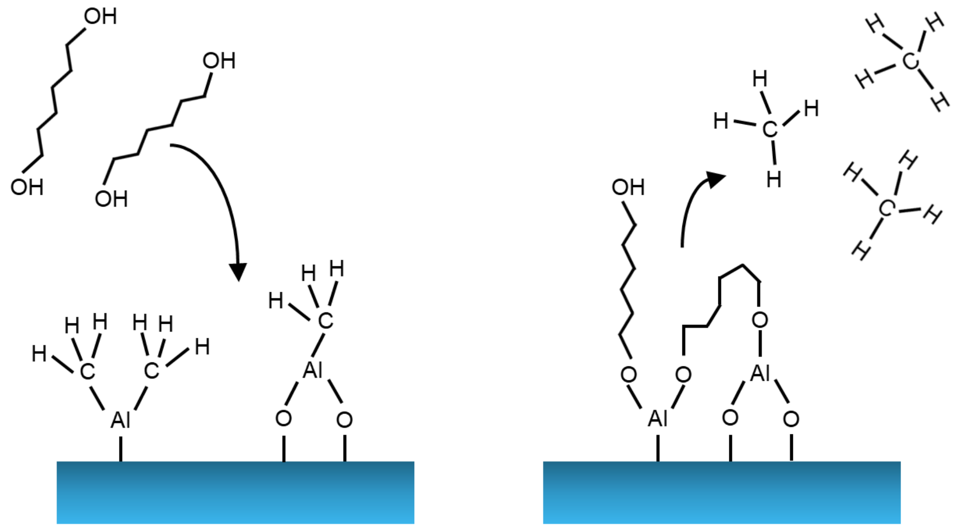

Figure S3: Long connected alucone film

S5. Picosecond acoustics of long connected alucone films

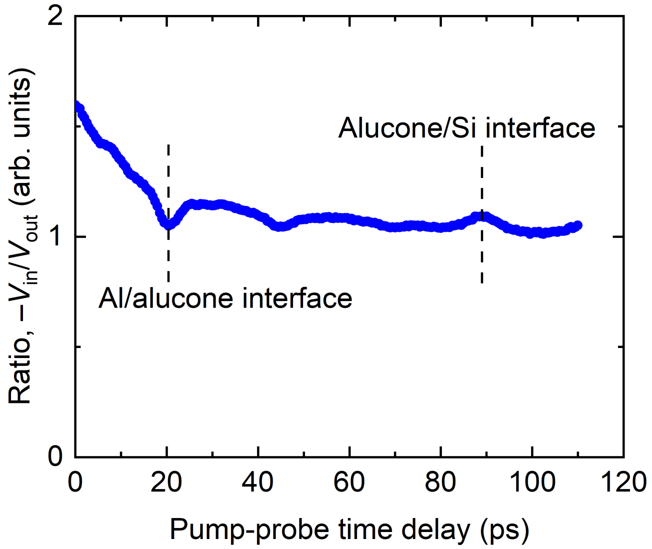

Figure S4: Picosecond acoustic response of the 156 nm long connected alucone film.

S6. Infrared spectroscopic ellipsometry of alucone films

Figures S5 and S6 show the ellipsometric data and model fits for the ordered and disordered alucone films, respectively. The angles used for the model fits correspond to the angles at which the ellipsometric measurements are performed. For the ordered and disordered samples, different angles are used during the measurements, chosen arbitrarily within the 50–75° range. The resulting optical properties are independent of the incident angle, and the model provides a good fit at any of these angles.

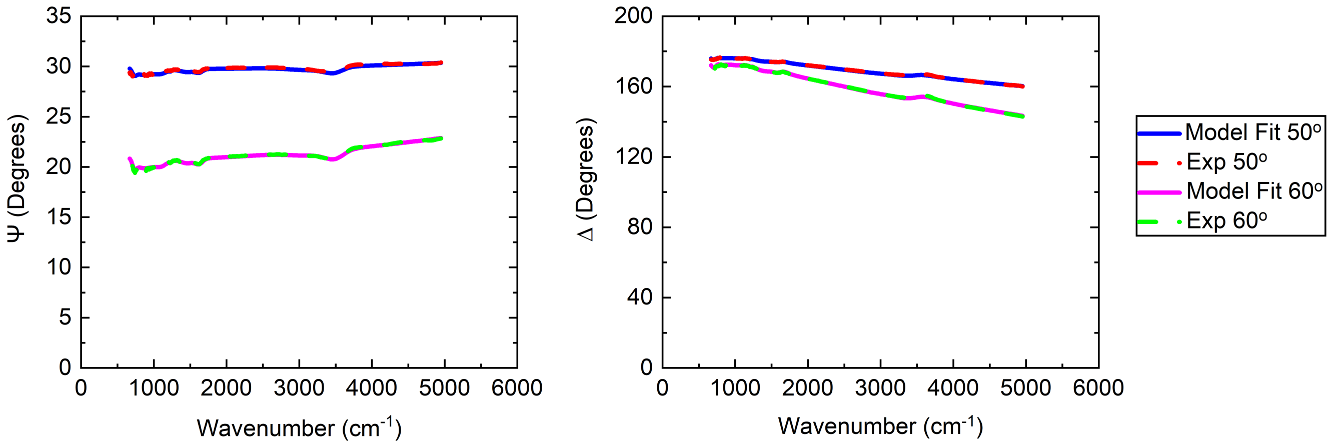

Figure S5: Measured data and model fits for the ordered alucone film.

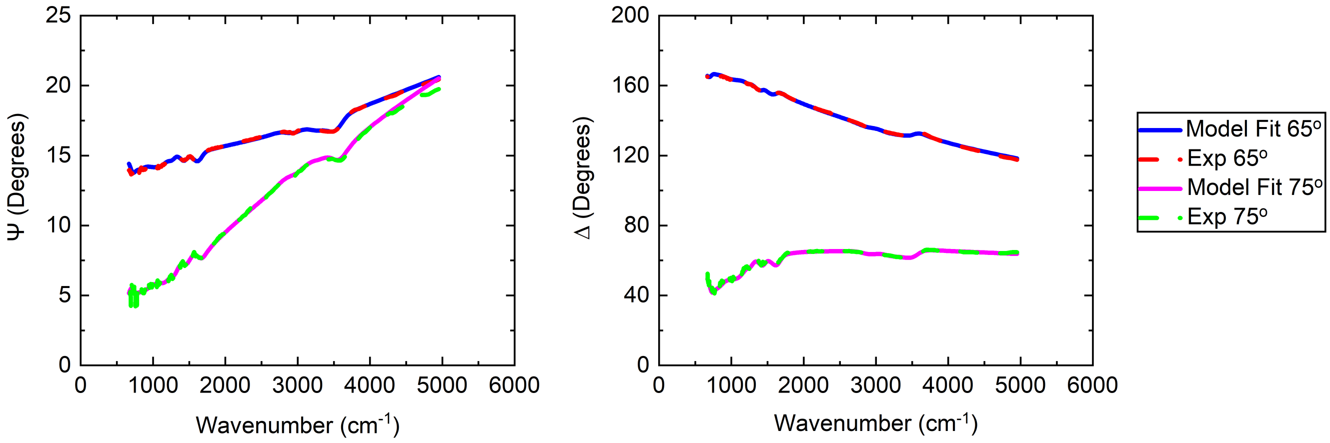

Figure S6: Measured data and model fits for the disordered alucone film.

Table S1: Gaussian oscillator parameters used to fit the short connected alucone film data, resulting in a mean square error of 1.36.

Oscillator No.	Amplitude	Centroid Frequency (cm^{-1})	Centroid Wavelength (um)	Broadening (cm^{-1})	Lifetime (fs)
1	0.19	3439.7	2.91	310.0	17.13 ± 1.82
2	0.13	3110.1	3.22	606.6	8.75 ± 1.52
3	0.22	1607.6	6.22	127.2	41.75 ± 3.04
4	0.26	1441.1	6.94	201.0	26.42 ± 2.43
5	0.07	1224.6	8.17	26.7	198.81 ± 96.13
6	0.45	1030.8	9.70	253.8	20.92 ± 3.61
7	0.85	791.65	12.63	234.4	22.65 ± 7.93
8	2.12	667.83	14.97	99.6	53.31 ± 6.74

Table S2: Gaussian oscillator parameters used to fit the long connected alucone film, resulting in a mean square error of 0.37.

Oscillator No.	Amplitude	Centroid Frequency (cm^{-1})	Centroid Wavelength (um)	Broadening (cm^{-1})	Lifetime (fs)
1	0.11	3487.6	2.87	220.9	24.03 ± 7.32
2	0.15	3259.1	3.07	352.8	15.05 ± 11.41
3	0.08	2912.4	3.43	231.6	22.92 ± 5.57
4	0.04	2578.4	3.88	960.3	5.53 ± 1.73
5	0.23	1590.7	6.29	148.9	35.66 ± 1.38
6	0.21	1414.2	7.07	107.8	49.26 ± 2.83
7	0.07	1259.6	7.94	81.1	65.43 ± 12.79
8	0.36	995.6	10.04	303.6	17.48 ± 2.1
9	0.46	756.3	13.22	189.6	28.01 ± 14.41
10	1.04	667.8	14.98	100.2	52.99 ± 10.37

Table S3: Thermal conductivity and longitudinal sound speed of the polymers shown in Figure 2(d).

Polymers	Thermal conductivity ($W\ m^{-1}\ K^{-1}$)	Longitudinal sound speed ($m\ s^{-1}$)
PVA	0.31	3210[4]
PAA	0.37	3740[4]
PVP	0.27	3180[4]
PAM	0.38	4340[4]
PSS	0.38	3640[4]
MC	0.21	2770[4]
PMMA	0.20	2850[4]
PAP	0.16	2640[4]
PALi	0.55	5100[5]
PANa	0.45	4100[5]
PACa	0.49	4800[5]
PAFe	0.51	5000[5]
PACu	0.5	4800[5]
PVPA	0.44	3900[5]
PVPLi	0.63	5300[5]
PVPMg	0.66	5400[5]
PVPCa	0.67	5300[5]
PVSNa	0.42	4300[5]
PDDA	0.29	3700[5]
PAH	0.34	4300[5]
PMPC	0.21	3300[5]
PS	0.141	2380[5]
ADP	0.15	2850[5]
DSQ	0.14	2100[5]
Vectra	1.7	6879[6]
Kevlar	3.2	10203[6]
M5 AS	8.9	9772[6]
Spectra 900	11.5	10275[6]
Spectra 2000	16	12343[6]
Dyneema	14.2	11404[6]
PBT	12.7	13929[6]
Zylon AS	18.5	12187[6]
Zylon HM	23	14850[6]

References

[1] Yang, W.; Hullavarad, S.; Nagaraj, B.; Takeuchi, I.; Sharma, R.; Venkatesan, T.; Vispute, R.; Shen, H. Compositionally-tuned epitaxial cubic Mg x Zn 1- x O on Si (100) for deep ultraviolet photodetectors. *Applied Physics Letters* **2003**, *82*, 3424–3426.

[2] Baek, G.; Lee, S.; Lee, J.-H.; Park, J.-S. Air-stable alucone thin films deposited by molecular layer deposition using a 4-mercaptophenol organic reactant. *Journal of Vacuum Science & Technology A* **2020**, *38*, 022411.

[3] Nye, R. A.; Kelliher, A. P.; Gaskins, J. T.; Hopkins, P. E.; Parsons, G. N. Understanding molecular layer deposition growth mechanisms in polyurea via picosecond acoustics analysis. *Chemistry of Materials* **2020**, *32*, 1553–1563.

[4] Xie, X.; Li, D.; Tsai, T.-H.; Liu, J.; Braun, P. V.; Cahill, D. G. Thermal conductivity, heat capacity, and elastic constants of water-soluble polymers and polymer blends. *Macromolecules* **2016**, *49*, 972–978.

[5] Xie, X.; Yang, K.; Li, D.; Tsai, T.-H.; Shin, J.; Braun, P. V.; Cahill, D. G. High and low thermal conductivity of amorphous macromolecules. *Physical Review B* **2017**, *95*, 035406.

[6] Wang, X.; Ho, V.; Segalman, R. A.; Cahill, D. G. Thermal conductivity of high-modulus polymer fibers. *Macromolecules* **2013**, *46*, 4937–4943.
